# Cannabinoids in the Brain: New Vistas on an Old Dilemma

**DOI:** 10.1155/2016/9146713

**Published:** 2016-05-24

**Authors:** Maurice Ptito, Christian Casanova, Jean-François Bouchard

**Affiliations:** ^1^École d'Optométrie, Université de Montréal, Montréal, QC, Canada H3T 1P1; ^2^Laboratory of Neuropsychiatry-Psychiatric Centre Copenhagen and BrainLab, University of Copenhagen, Copenhagen, Denmark

The use of cannabis as a therapeutic and recreational substance goes back to thousands of years throughout Asia, Middle East, Southern Africa, and South America. The discovery of Δ-9-tetrahydrocannabinol (Δ9-THC) by Mechoulam and Gaoni in the midsixties as the major psychoactive constituent of cannabis sativa [1, 2] led to another important discovery, namely, its specific binding site that was isolated and cloned in 1990 [3]. This first cannabinoid receptor was coined CB1R and triggered a number of investigations on its expression, localization, and function within the body tissue including the brain, in various species. This was followed by the discovery in 1992 of the first endocannabinoid (eCB), anandamide [4], followed by another cannabinoid receptor CB2R and a second endocannabinoid called 2-arachidonoylglycerol (2-AG) [5, 6]. Later on, some of the enzymes responsible for their synthesis (*N-acyl* phosphatidylethanolamine phospholipase D (NAPE-PLD); diacylglycerol lipase (DAGL)) and degradation (fatty acid amide hydrolase (FAAH); monoacylglycerol lipase (MAGL)) were identified.

The recreational use and side effects of cannabinoids have been subjected to numerous studies throughout history and the list of neurobehavioral effects of chronic use of marijuana is long and well known. For example, significant impairments have been reported on the visual system (e.g., photosensitivity, depth perception, color, motion, scotopic, and photopic functions) [7, 8], the motor system (movement control and coordination in driving) [9, 10], motivations (e.g., increased hunger known as “munchies”) [11], higher cognitive functions (reward, cognition, learning, and memory) [12], and emotions (e.g., fear, depression, anxiety, and paranoia) [13] have been reported. However, the investigation of the endocannabinoid (eCB) system and its potential therapeutic use is rather new, and a lot of attention has been devoted not only to its specific expression in the brain but also to its functions. Studies on the expression and localization of the cannabinoid receptors in the brain have burgeoned in the last decade and have furnished valuable data on their putative involvement in various sensory-motor and cognitive functions in diverse animal species, including Man. These studies have recently received substantial attention from pharmaceutical companies as a potential source for novel treatments. Additionally, the dilemma of legalizing the use of cannabis in some countries makes the investigation on cannabinoid systems more momentous. This special issue is therefore timely and brings historical and groundbreaking novel research on the role of these cannabinoid receptors in the mammalian central nervous system (CNS).

In this issue, we present a number of review papers and original articles on the eCB system ranging from vision to cognition in mice, rats, tree shrews, monkeys, and humans. Cannabinoid exposure during adolescence increases the effects of certain drugs [14]. In their original article, M. Rodríguez-Arias et al. demonstrate that exposure of adolescent male mice to WIN 55212-2 (a cannabinoid agonist) increases the cocaine rewarding effects. They also conclude that the development of drug addiction is dependent on the propensity for sensation-seeking. Heavy and prolonged cannabis consumption by teenagers could as well produce a wide range of structural and functional modifications in the brain [15, 16]. In their research paper, S. J. Broyd and colleagues evaluate the recovery of mismatch negativity and the P50 sensory gating in cannabis ex-users. They conclude that the modifications induced by cannabis usage during adolescence are persistent even after a prolonged period of abstinence.

Until recently, the vast majority of the cannabinoid effects in the CNS were attributed exclusively to the CB1R. However, recent evidence also demonstrates the presence of the CB2R in the nervous system (for review, see [17]). Using CB2R knockout (KO) mice, Y. Li and J. Kim assess the role played by the CB2R in memory. They demonstrate that, depending on the memory type, CB2R plays different roles. For example, CB2R affects preferentially the hippocampus-dependent memory such as long-term contextual fear memory. They also show that the CB2R KO mice have an enhanced working memory.

Two original research articles on the retina are proposed by the Montreal group. In the first one, the authors provide interesting data on interspecies comparisons of the distribution and localization of cannabinoid receptors. Since most of the data, to this day, has been collected on rodents, J. Bouskila and collaborators provide results on four species along the phylogenetic scale and show major differences between them. The expression of CB1R, FAAH, MAGL, and DAGL is similar for all species whereas CB2R and NAPE-PLD are differently expressed. In monkeys, NAPE-PLD is restricted to the photoreceptor layer and CB2R is found in Müller cells only. In the second paper, J. Bouskila et al. propose a functional role of CB1R and CB2R in primate vision using electroretinographic recordings (ERG). They show that blockade of either CB1R or CB2R by the intravitreal injection of specific antagonists has differential effects. In photopic conditions, blocking CB1R increases the amplitude of the a-wave of the ERG whereas blocking CB2R increases both a- and b-waves.

The papers by J.-F. Bouchard et al. and T. Schwitzer et al. provide extensive and up-to-date reviews on cannabinoid research. In their review, J.-F. Bouchard et al. characterize the expression and physiological functions of the eCB system in the visual system, from the retina to the primary visual cortex. They report that eCB system is widely present in the retina, where it acts as a modulator of neurotransmitter release and ion channel activity. T. Schwitzer et al., besides reviewing the distribution of the eCB system throughout the retina, describe the crucial role of the cannabinoid system in neurotransmission, neural plasticity, and neuroprotection. Both reviews highlight the potential use of cannabinoids as therapeutic pharmacological agents for the protection and treatment of retinal diseases.

Retinal diseases as glaucoma receive a particular attention in the next two reviews. E. A. Cairns et al. review the evidence of eCB as a potential target for the treatment of glaucoma and propose that this system has also a neuroprotective function. D. Kokona et al. present, besides glaucoma, research on other retinal pathologies such as diabetic retinopathy. These retinal neurodegenerative diseases lead to massive retinal neuron loss and lead to blindness. The neuroprotective putative role of eCB is also highlighted here.

In order to study the physiological and the pathophysiological effects of eCBs (anandamide, 2-AG, PEA, etc.) in the CNS, we need to quantify their levels. In their article, J. Keereetaweep and K. D. Chapman review the recent and sensitive mass spectrometry methods allowing the development of lipidomic approaches and methodologies to quantify these lipid mediators in the CNS.

We hope that the collected papers in this special issue will contribute to the understanding of the various mechanisms involved in the functions of the endocannabinoid system and the development of new pharmaceutical tools to treat visual disorders.
